# On the scattering of torsional waves from axisymmetric defects in buried pipelines

**DOI:** 10.1121/1.4983192

**Published:** 2017-05-16

**Authors:** Wenbo Duan, Ray Kirby, Peter Mudge

**Affiliations:** 1 Department of Mechanical, Aerospace and Civil Engineering, Brunel University London, Uxbridge, Middlesex, UB8 3PH, United Kingdom; 2 Integrity Management Group, TWI Ltd, Cambridge, CB21 6AL, United Kingdom

## Abstract

This article develops a numerical model suitable for analysing elastic wave scattering in buried pipelines. The model is based on a previous so-called hybrid approach, where a nominally infinite length of pipe is split up into uniform and non-uniform regions. The key challenge for buried structures is in enforcing the appropriate boundary conditions in both the axial and radial directions, which must encompass the entire length of the structure, as well as the surrounding material. Accordingly, the focus of this article is on developing a model suitable for accurately applying these boundary conditions, and so the analysis is restricted here to the study of axisymmetric defects and to an incident sound field that consists of the fundamental torsional mode only. It is shown that this problem may be addressed in a numerically efficient way provided one carefully choses a perfectly matched layer for the surrounding material, and then integrates over this layer using a complex co-ordinate stretching function. This enables the use of mode matching to deliver a convergent system of equations that enforce the appropriate axial and radial boundary conditions.

## I. INTRODUCTION

Elastic waves are often used in the non-destructive evaluation of structures. Normal practice is to use a pulse-echo technique and in order to identify fabrication or in-service defects one must analyse and interpret the returning echo in the time domain. For long slender structures, such as pipelines or rails, a technique known as Long Range Ultrasonic Testing (LRUT) is popular, as it uses lower ultrasonic frequencies and ultrasonic wave modes with inherently low attenuation properties to penetrate long sections of a structure.^1^ However, structures such as pipelines support the propagation of many different guided wave eigenmodes and the interpretation of echoes returning from a defect presents many challenges. Furthermore, it is common for pipelines to be buried underground and this further complicates the propagation, and hence interpretation, of pipe eigenmodes. It is, therefore, desirable to develop theoretical models in order to obtain a good understanding of how elastic waves scatter in buried structures, before then investigating ways to improve the effectiveness of LRUT. Accordingly, this article presents a theoretical model suitable for analysing scattering from an axisymmetric defect in a buried, or embedded, pipeline. The theoretical model is based on a hybrid numerical method that solves the problem in a computationally efficient way; this enables the generation of theoretical predictions for a problem that is likely to require a prohibitive number of degrees of freedom to solve when using commercial finite element based software.

The theoretical investigation of guided waves in structures such as pipelines is now well established, although the majority of articles focus on computing the propagating eigenmodes. This may be achieved by solving analytic expressions written in matrix form, see for example the popular software Disperse,^2,3^ or by using numerical methods such as the Semi Analytic Finite Element (SAFE) method.^4^ The computation of eigenmode properties for a structure provides important information for use in LRUT, with properties such as modal energy velocity enabling the user to distinguish between different modes through time of flight calculations. However, solving the eigenproblem does not provide information regarding the amplitude of each scattered mode when the waveguide incorporates non-uniform regions. This is a different challenge, as one must move from the analysis of an infinite waveguide to a finite, or more usually semi-infinite, guide. This is especially problematic in the analysis of large structures such as pipelines, where under favourable conditions the inspection range can extend to well over 10 m. For example, in a recent study Leinov *et al.*^5^ required 21.13 million hexahedral elements to study a partially embedded pipe that was 4 m long. This is likely to deliver over 100 million degrees of freedom for this pipe, and even with such a large number, the upper centre frequency was restricted to 35 kHz. Cleary this is not practical approach for larger systems, higher frequencies and/or fully buried pipes, especially in view of problems that are likely to be encountered with numerical dispersion with such large discretisation schemes. This means that commercial finite element based software is likely to find it very difficult to address a fully buried system, and to the best of the authors' knowledge no solutions for this type of problem are currently available. Accordingly, alternative methods are required if long lengths of fully buried systems are to be studied and so the development of a new computationally efficient approach forms the subject of this current article.

This article focusses on the development of a numerical model for the analysis of pipes that are buried, or completely embedded, in an elastic material that extends to infinity as one moves away from the pipe. That is, the energy radiated outward from the pipe into the surrounding elastic medium is not reflected back toward the pipe. In general FE formulations the application of this non-reflecting boundary condition requires a numerical approximation to be developed and common methods include absorbing layers,^6^ or the popular perfectly matched layers (PMLs).^7^ However, this means that a PML must surround the entire length of the pipe, and clearly this would significantly increase the degrees of freedom required to study this problem. It would also present significant problems associated with choosing an appropriate PML that minimises numerical noise in three dimensions. Accordingly, alternative methods are required that are far more computationally efficient, and here the authors favour a so-called hybrid numerical approach that has been developed for unburied structures. This method couples an eigensolution for a uniform region to a full FE discretisation of a region closely surrounding a defect. This method is much faster than a full FE discretisation, although it does depend on the assumption that the structure consists of long uniform lengths and relatively small non-uniform defects. Fortunately, this is normally the case in problems such as non-destructive testing in pipelines and so this method provides a practical way of systematically reducing the size of a computational model used to study LRUT in pipelines. The basic methodology for this hybrid method has been around for many years and is reported in a number of articles that examine defects in a solid cylinder (for example, see Ref. 8). The hybrid method has also recently been reported for pipelines by Duan *et al.*,^9,10^ and here it is shown that the efficiency of the method enables the analysis of long lengths of coated and uncoated pipes in both the time and the frequency domain. However, this hybrid method has not yet advanced as far as the analysis of buried or embedded structures, and given the large number of applications of buried pipelines there is a clear need to advance this approach so that it is capable of addressing the scattering problem for buried pipelines.

The analysis of guided wave propagation in buried structures has to date followed the practice seen for the early studies of unburied structures, so that analysis has focussed on the computation of eigenmodes. For example, low order axisymmetric modes may be obtained at ultrasonic frequencies using a matrix based analytic technique,^11^ as well as at low audio frequencies using limiting values.^12,13^ The SAFE method may also be applied to buried structures and here Castaings and Lowe^6^ applied the method to a structure of arbitrary cross-section. Castaings and Lowe used an artificial absorbing layer to represent the surrounding medium, and the properties of this layer are chosen to enforce of the radial boundary condition at infinity. However, absorbing layers have been shown to be relatively inefficient for this type of problem and more efficient approaches have recently been developed using PMLs.^7^ For example Nguyen *et al.*^7^ introduced a two dimensional PML to extract eigenmodes for a structure of arbitrary cross-section, and Treyssède^14^ further improved the efficiency of the method by using spectral elements. Duan *et al.*^15^ recently introduced a one dimensional approach for buried pipelines by taking advantage of the symmetric nature of this particular problem, which enabled modes to be extracted in a fast and efficient way. Additional methods may also be found in the literature for solving this eigenproblem, and a more comprehensive review is provided in the article by Duan *et al.*^15^ These methods are, however, limited to solving the eigenproblem for buried pipes and this analysis has yet to be extended to address the full scattering problem associated with defects in slender structures such as pipelines.

Solving the scattering problem for buried structures presents additional problems related to fulfilling the radial boundary condition in the surrounding material. For this reason there are very few articles that address scattering in buried, or embedded, cylindrical structures of finite or semi-finite length. The recent work of Leinov *et al.*^5^ examined a partially buried pipe, whereas for fully buried systems that avoid a full FE discretisation, previous work has generally been restricted to viscometers, where guided waves are used to infer the properties of a surrounding medium. Relevant examples for viscometers include the use of longitudinal modes to measure the viscosity of different fluids,^16^ as well as the measurement of density^17,18^ and the dependence of properties on temperature.^19^ These studies, and many other related articles in this area, all rely on a combination of the computation of eigenmodes and experimental measurement. It is only Vogt *et al.*^20^ who go further and develop a theoretical model to capture the reflection of elastic waves from the junction between the bulk fluid and the viscometer. Vogt *et al.* found it necessary to develop a theoretical model to capture the scattering from the bulk fluid because they were measuring the properties of epoxy resin during curing. The resin generates significant reflections and it was found to be necessary to try and quantify this in order to interpret experimental measurements. Accordingly, Vogt *et al.* developed an analytic approach based on mode matching to obtain a scattering matrix for the junction between the two regions. However, in order to obtain this matrix the authors enforce the axial continuity conditions over the cylinder only, and so do not enforce the traction free boundary condition over the vertical surface of the embedding medium. This approximation does have the advantage of removing the singularity in the stress field at the corner of the step change where the cylinder becomes immersed in the epoxy resin. Removing this singularity means that one may readily obtain a convergent system of equations using only a small number of propagating modes, and so Vogt *et al.* were able to apply their technique with some success. However, the method only provides an approximation of the axial matching conditions, and if one seeks also to include the appropriate boundary condition over the free surface of the resin then any mode matching technique must also accommodate the singularity in the stress field at the corner where the free surface meets the cylinder. It is possible to do this using mode matching, however this normally requires additional evanescent modes in order to enforce accurately the axial matching conditions, and the number of evanescent modes required is related to the strength of the singularity.^21^ Thus, the method of Vogt *et al.*^20^ is only an approximation of the true scattering problem and it is not clear how accurate such an approach is likely to be when applied to different problems, or to different parameters for a similar problem. Furthermore, the method of Vogt *et al.* relies on a uniform geometry to be present on either side of the step change, and so their mode matching technique is not suited to the study of non-uniform scattering problems, such as those associated with cracks or corrosion. Accordingly, there is a need to move on from analytic mode matching techniques when analysing more complex scattering problems, and so this article extends the hybrid approach seen previously for unburied structures^8–10^ and applies it to the study of scattering in buried structures.

The extension of a hybrid SAFE-FE modelling approach to buried structures is, however, far from straightforward because of difficulties associated with the enforcement of the appropriate boundary conditions over a semi-infinite domain. Furthermore, Vogt *et al.*^20^ raise questions regarding modal orthogonality and the impact this may have on a mode matching based solution, which normally underpins a hybrid based approach. Therefore, this remains a complex problem and in order to focus on key issues relating to modal orthogonality and the enforcement of relevant boundary and matching conditions, this article will focus on the scattering of the fundamental [shear] torsional mode T(0,1) from an axisymmetric defect in a pipe. The article begins by briefly recapping on the eigenproblem for a buried pipe, and then introduces a hybrid modal-FE model in Sec. II. The accuracy of the new approach, including the issue of modal orthogonality and mode matching, is examined in Sec. III; and additional results are also presented in Sec. IV to illustrate some practical problems that may occur when undertaking LRUT in the field; conclusions are then drawn in Sec. V.

## II. THEORY

**FIG. 1.**
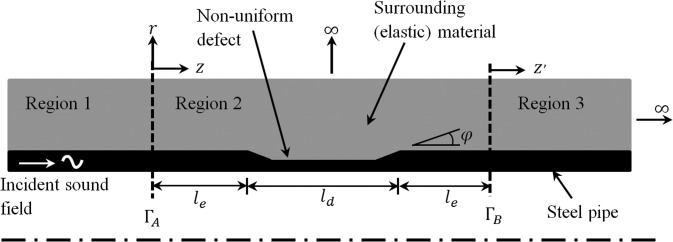
Geometry of non-uniform defect in a buried pipeline.

The geometry of an axially non-uniform axisymmetric defect is shown in Fig. 1. Here, the term ‘non-uniform’ means that the geometry of the defect changes in the axial direction, which is illustrated using tapering of the defect in Fig. 1. The defect is placed in a semi-infinite pipeline that consists of three regions: regions 1 and 3 are axisymmetric and uniform, and region 2 is axisymmetric and this encloses the non-uniform defect. The pipe is buried so that each region is embedded by an elastic material, so that this material is in contact with the surface of the pipe as well as the defect. Each region is joined to an adjacent region by a vertical interface, so that $\Gamma_{A}$ separates regions 1 and 2, and $\Gamma_{B}$ separates regions 2 and 3. The hybrid SAFE-FE approach is adopted here so that SAFE modal solutions are sought for regions 1 and 3, and a full finite element discretisation is adopted for region 2. Mode matching is used to enforce the appropriate continuity conditions over each vertical interface.

### A. Eigensolution for a uniform region

To maintain generality the buried pipe is assumed to have an arbitrary number of layers that may have different material properties (for example, a viscoelastic coating), as well a PML layer that provides the outer boundary and is used to enforce the appropriate radiation boundary condition. The pipe substrate is numbered j=1, additional layers are numbered j=2 to m−1, and the outer PML layer is numbered j=m. A SAFE-PML technique for this geometry was recently presented by Duan *et al.*,^15^ and this can be used to calculate all axisymmetric and non-axisymmetric (flexural) modes. However, in this study we will simplify the analysis of Duan *et al.* and focus on the torsional modes only. This means that the governing equation for the waveguide in region 1, may be reduced to^15^

$$\rho_{j}\frac{\partial^{2}u_{1\theta_{j}}^{\prime }}{\partial t^{2}}=\frac{\partial \sigma_{1\theta r_{j}}^{\prime }}{\partial r}+\frac{\partial \sigma_{1\theta z_{j}}^{\prime }}{\partial z}+\frac{2}{r}\sigma_{1r\theta_{j}}^{\prime },$$ (1)

where *r*, θ and *z* form an orthogonal cylindrical co-ordinate system in the radial, circumferential and axial direction of the waveguide, respectively; ρ is density, t is time, $u_{1\theta }^{\prime }$ is the torsional displacement in region 1, and $\sigma_{1\theta r}^{\prime }$ and $\sigma_{1\theta z}^{\prime }$ are shear stresses. A time dependence of $e^{i\omega t}$ is assumed throughout this article, where ω is the radian frequency and $i=\sqrt{-1}$. The torsional displacement is expanded as an infinite set of eigenmodes so that for any mode, *n*,

$$u_{1\theta }^{\prime }\left(r,\;z\right)=u_{1\theta }\left(r\right)e^{-ik\gamma z},\;$$ (2)

where $u_{1\theta }(r)$ is the eigenfunction, and γ the dimensionless wavenumber. In addition, $k=\omega /c_{T}$, and $c_{T}$ is the shear (torsional) bulk wave velocity of the substrate pipe (layer j=1). In the outer layer j=m, the radial coordinate r is replaced by a stretched coordinate $\tilde{r}$, which is defined as

$$\tilde{r}=\int_{0}^{r}\xi_{r}(s)\;\text{ds}.$$ (3)

Here, $\xi_{r}$ is a non-zero, continuous and complex-valued coordinate stretching function, which defines the PML. The function proposed by Duan *et al.*^15^ is adopted here, so that

$$\xi_{r}\left(r\right)=e^{\alpha \bar{r}}-i\left[e^{\beta \bar{r}}-1\right],$$ (4)

where $\bar{r}=(r-a_{m})/h$, and the thickness of the PML layer is $h=b_{m}-a_{m}$. In addition, α and β are real valued constants. Following the analysis of Duan *et al.*,^15^ Galerkin's method is used to derive the weak form of the governing equation, and so integration by parts yields

$$\begin{array}{c} \int_{a_{j}}^{b_{j}}\mu_{j}\{\frac{1}{\xi_{r}}\frac{\partial w_{1\theta_{j}}}{\partial r}\frac{\partial u_{1\theta_{j}}}{\partial r}-\frac{\partial w_{1\theta_{j}}}{\partial r}\frac{u_{1\theta_{j}}}{\tilde{r}}-2\frac{w_{1\theta_{j}}}{\tilde{r}}\frac{\partial u_{1\theta_{j}}}{\partial r}+2\xi_{r}w_{1\theta_{j}}\frac{u_{1\theta_{j}}}{{\tilde{r}}^{2}}+\xi_{r}\left[k^{2}\gamma^{2}-\frac{\rho_{j}\omega^{2}}{\mu_{j}}\right]w_{1\theta_{j}}u_{1\theta_{j}}\}\;dr=w_{1\theta_{j}}\sigma_{1\theta r_{j}}{|}_{b_{j}}-w_{1\theta_{j}}\sigma_{1\theta r_{j}}{|}_{a_{j}}, \end{array}$$ (5)

where for j=1, m−1, $\xi_{r}=1$ and $\tilde{r}=r$. In addition, μ is a Lamé coefficient, and $a_{j}$ and $b_{j}$ are the inner and outer radius of layer j. Finite element discretisation in the radial direction only yields

$$u_{1\theta }\left(r\right)=\sum_{p=1}^{p_{\theta }}N_{\theta p}\left(r\right)u_{1\theta p}=N_{1\theta }u_{1\theta },$$ (6)

where $N_{\theta }$ is a global trial (or shape) function, $u_{1\theta p}$ is the value of $u_{1\theta }$ at node p, and $p_{\theta }$ is the number of nodes (or degrees of freedom) used to discretise the radial geometry. In addition, $N_{1\theta }$ and $u_{1\theta }$ are row and column vectors of length $p_{\theta }$, respectively. Isoparametric elements are used so the weighting functions $W_{1\theta }=N_{1\theta }$.

The appropriate boundary conditions that join together layers in the problem are continuity of displacement and shear stress, as well as a traction free boundary condition that closes the problem at the inner surface of the pipe, $r=a_{1}$. The choice of boundary condition on the outer surface of the PML layer is arbitrary because a PML damps down outward propagating waves.^7^ Therefore, it is convenient to choose a traction free boundary condition to close the outer surface of the PML at $r=b_{m}$, as this permits a simplification of the equations that follow. After applying these boundary conditions to each layer, the following eigenequation is obtained:

$$(Z_{1\theta }+Z_{1m})u_{1\theta }=-k^{2}\gamma^{2}[\Sigma_{j=1}^{m}\mu_{j}K_{4j}]u_{1\theta }.$$ (7)

The matrices that make up this equation are listed in Appendix A. Equation (7) is a sparse eigenequation and solution of this equation will deliver an unordered list of $p_{\theta }$ eigenmodes. This equation is solved here using the sparse eigensolver “eigs” in matlab. This delivers an unordered list of $p_{\theta }$ eigenvalues, γ, and associated eigenvectors,  Ф. This then enables the displacement in each uniform region to be expressed as a sum of these eigenmodes, so that

$$u_{1\theta }\left(r\right)=\sum_{n=0}^{m_{1}}A^{n}{Ф}_{\theta +}^{n}(r)e^{-ik\gamma^{n}z}+\sum_{n=0}^{m_{1}}B^{n}{Ф}_{\theta -}^{n}(r)e^{ik\gamma^{n}z},$$ (8)

$$u_{\theta 3}\left(r\right)=\sum_{n=0}^{m_{3}}C^{n}\Psi_{\theta +}^{n}(r)e^{-ik\beta^{n}z^{\prime }}.$$ (9)

Here, $A^{n}$, $B^{n}$ and $C^{n}\;$ are modal amplitudes, ${Ф}_{\theta +}^{n}$ and ${Ф}_{\theta -}^{n}$ are eigenvectors for the incident and reflected waves in region 1, respectively. In region 3, it is convenient to use a different notation so that β is the dimensionless eigenvalue and $\Psi_{\theta +}^{n}$ is the eigenvector. Finally, these infinite sums are truncated at mode $m_{1}$ in region 1, and $m_{3}$ in region 3.

### B. FE discretisation of the non-uniform region

**FIG. 2.**
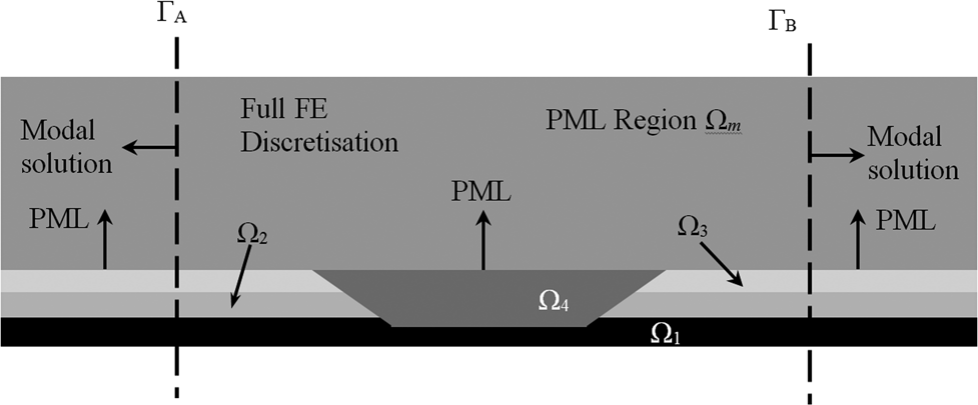
A two dimensional finite element discretisation is applied to the central region, between Γ_A_ and Γ_B_. Here, two additional layers are shown, and each area $\Omega_{j}$ (j=1,2,3,4,m) is bounded by the surface $\Gamma_{j}$, so that $\Gamma_{j}$ does not extend beyond the vertical boundaries Γ_A_ and Γ_B_ and areas $\Omega_{j}$ (j=1,2,3,m) all include a portion of Γ_A_ and Γ_B_.

Region 2 encloses an axisymmetric defect of arbitrary geometry. Accordingly, a conventional two dimensional finite element discretisation is applied here and this is coupled to a PML that abuts onto the pipe, see Fig. 2. That is, the conventional finite element discretisation encompasses the pipe wall $\Omega_{1}$, any additional layers (e.g., $\Omega_{2}$ and $\Omega_{3}$), and the region abutting the defect that does not extend beyond the outer radius of the additional layers, which is denoted here $\Omega_{4}$. The PML starts at the radius of the outer layer of the pipe and this region is denoted $\Omega_{m}$ in Fig. 2. The weak form of Eq. (1) is obtained using the weighting function $w_{2}$, and then integrating by parts in the radial and axial directions separately. This separation of variables allows the PML to be applied in the radial direction only and so for layer j, with for j=1 to m−1, this yields

$$\begin{array}{c} \int_{\Omega_{j}}\mu_{j}\left\{\frac{\partial w_{2j}}{\partial r}\frac{\partial {u^{\prime }}_{2j}}{\partial r}+\frac{\partial w_{2j}}{\partial z}\frac{\partial {u^{\prime }}_{2j}}{\partial z}-\frac{\partial w_{2j}}{\partial r}\frac{{u^{\prime }}_{2j}}{r}-\frac{2}{r}w_{2j}\frac{\partial {u^{\prime }}_{2j}}{\partial r}+2w_{2j}\frac{{u^{\prime }}_{2j}}{r^{2}}-\frac{\rho_{j}\omega^{2}}{\mu_{j}}w_{2j}{u^{\prime }}_{2j}\right\}d\Omega_{j}=\int_{\Gamma_{j}}w_{2j}{\sigma^{\prime }}_{2r\theta_{j}}n_{r}d\Gamma_{j}+\int_{\Gamma_{j}}w_{2j}{\sigma^{\prime }}_{2\theta z_{j}}n_{z}d\Gamma_{j}. \end{array}$$ (10)

Here, $\Omega_{j}$ denotes the two dimensional area of layer j, and $\Gamma_{j}$ is the outer boundary of $\Omega_{j}$. Application of the PML in the radial direction only for layer j=m yields

$$\begin{array}{c} \int_{\Omega_{m}}\mu_{m}\{\frac{1}{\xi_{r}}\frac{\partial w_{2m}}{\partial r}\frac{\partial {u^{\prime }}_{2m}}{\partial r}+\xi_{r}\frac{\partial w_{2m}}{\partial z}\frac{\partial {u^{\prime }}_{2m}}{\partial z}-\frac{\partial w_{2m}}{\partial r}\frac{{u^{\prime }}_{2m}}{\tilde{r}}-\frac{2}{\tilde{r}}w_{2m}\frac{\partial {u^{\prime }}_{2m}}{\partial r}+2\xi_{r}w_{2m}\frac{{u^{\prime }}_{2m}}{{\tilde{r}}^{2}}-\frac{\rho_{m}\omega^{2}}{\mu_{m}}\xi_{r}w_{2m}{u^{\prime }}_{2m}\}d\Omega_{m}=\int_{\Gamma_{m}}w_{2m}{\sigma^{\prime }}_{2r\theta_{m}}\;n_{r}d\Gamma_{m}+\int_{\Gamma_{m}}\xi_{r}w_{2m}{\sigma^{\prime }}_{2\theta z_{m}}n_{z}d\Gamma_{m}. \end{array}$$ (11)

Finally, the circumferential displacement $u_{2\theta }^{\prime }\left(r,z\right)$ in region 2 is discretised to give

$$u_{2\theta }^{\prime }\left(r,z\right)=\sum_{p=1}^{p_{2}}N_{2p}\left(r,z\right)u_{2p}=N_{2}u_{2},$$ (12)

where $N_{2p}$ is a global shape function and $u_{2p}$ is the value of $u_{2\theta }^{\prime }$ at node *p*, and $p_{2}$ is the number of nodes in region 2.

### C. Application of boundary conditions

Equations (10) and (11) apply to individual layers and so to combine these layers together the appropriate boundary conditions must be enforced between them. The usual relationships between stress and strain apply, so that

$$\sigma_{2r\theta_{j}}^{\prime }=\mu_{j}\left(\frac{\partial u_{2j}^{\prime }}{\partial r}-\frac{u_{2j}^{\prime }}{r}\right)$$ (13)

and

$$\sigma_{2\theta z_{j}}^{\prime }=\mu_{j}\frac{\partial u_{2j}^{\prime }}{\partial z}.$$ (14)

Note that for the PML layer (j=m), the radial coordinate in Eq. (13) should be replaced by the complex coordinate stretching function.

To join each region together it is necessary to enforce the axial continuity conditions of displacement and normal shear stress over planes $\Gamma_{A}$ and $\Gamma_{B}$. The shear stress condition is enforced by substituting the modal expansions in Eqs. (8) and (9) into the integrals on the right hand sides of Eqs. (10) and (11). Note that the normal on planes $\Gamma_{A}$ and $\Gamma_{B}$ in the radial direction equals zero and so the first term on the right hand sides of Eqs. (10) and (11) vanishes. For the pipe and additional layers this yields

$$\begin{array}{c} \int_{\Gamma_{A}}w_{2j}\;{\sigma^{\prime }}_{2\theta z_{j}}n_{z}dr=ik\gamma^{n}\left[A^{n}\sum_{j=1}^{m-1}\int_{a_{j}}^{b_{j}}\mu_{j}w_{2j}\Phi_{\theta +}^{n}dr-B^{n}\sum_{j=1}^{m-1}\int_{a_{j}}^{b_{j}}\mu_{j}w_{2j}\Phi_{\theta -}^{n}dr\right] \end{array}$$ (15)

and

$$\int_{\Gamma_{B}}w_{2j}\sigma_{2\theta z_{j}}^{\prime }n_{z}dr=-ik\gamma^{n}C^{n}\sum_{j=1}^{m-1}\int_{a_{j}}^{b_{j}}\mu_{j}w_{2j}\Psi_{\theta +}^{n}\;dr,$$ (16)

for mode n. Note that the unit normal in region 2 points in the negative z direction on $\Gamma_{A}$, whereas it points in the positive *z* direction on $\Gamma_{B}$. These axial matching conditions must also be enforced over the outer PML, and this forms a crucial part of implementing the hybrid method for buried structures. The key to applying these axial conditions is to integrate over the stretched co-ordinate in the PML region, which for mode n gives

$$\begin{array}{c} \int_{\Gamma_{A}}\xi_{r}w_{2m}{\sigma^{\prime }}_{2\theta z_{m}}n_{z}dr=ik\gamma^{n}\left[A^{n}\int_{a_{m}}^{b_{m}}\mu_{m}\;\xi_{r}w_{2m}\Phi_{\theta +}^{n}\;dr-B^{n}\int_{a_{m}}^{b_{m}}\mu_{m}\;\xi_{r}\;w_{2m}\;\Phi_{\theta -}^{n}\;dr\right] \end{array}$$ (17)

and

$$\int_{\Gamma_{B}}\xi_{r}w_{2m}\sigma_{2\theta z_{m}}^{\prime }n_{z}dr=-ik\gamma^{n}C^{n}\int_{a_{m}}^{b_{m}}\mu_{m}\xi_{r}w_{2m}\Psi_{\theta +}^{n}\;dr.$$ (18)

Substitution of Eqs. (15) to (18) back into Eqs. (10) and (11), and the application of the boundary conditions between each layer in the radial direction (remembering that each unit normal points outward), enables the governing equations in region 2 to be written as

$$\left[K_{1g}-K_{2g}-2K_{2g}^{T}+2K_{6g}-\omega^{2}K_{4g}\right]u_{2}=Q_{1g+}A-Q_{1g-}B-Q_{2g+}C,$$ (19)

where A, B and C are column vectors holding the modal amplitudes $A^{n}$, $B^{n}$, and $C^{n}$ respectively. The matrices in these equations are given in Appendix B.

Continuity of displacement over planes $\Gamma_{A}$ and $\Gamma_{B}$ is enforced separately and in order to do this it is convenient to weight this matching condition.^9,10^ Accordingly, the weighting functions $ik\gamma^{l}{Ф}_{\theta -}^{l}$ and $ik\beta^{l}\Psi_{\theta +}^{l}$ are chosen here, and integrations for layers from j=1 to m−1 are carried out in the usual way; integration for the PML layer j=m  is carried out in the stretched coordinate in the same way as for the shear stress. This yields

$$u_{2\theta }^{\prime }(r,0)=\sum_{n=0}^{m_{1}}A^{n}{Ф}_{\theta +}^{n}\left(r\right)+\sum_{n=0}^{m_{1}}B^{n}{Ф}_{\theta -}^{n}(r)$$ (20)

and

$$u_{2\theta }^{\prime }\left(r,L\right)=\sum_{n=0}^{m_{3}}C^{n}\Psi_{\theta +}^{n}\left(r\right),$$ (21)

where $L=2l_{e}+l_{d}$. Application of the weighting functions and integration over cross-sectional planes $\Gamma_{A}$ and $\Gamma_{B}$, yields

$$-Q_{3g-}^{T}u_{2}+M_{1\theta -}B=-M_{1\theta +}A$$ (22)

and

$$Q_{4g+}^{T}u_{2}-M_{3\theta +}C=0.$$ (23)

The matrices that make up these two equations are reported in Appendix B. The question of modal orthogonality is relevant to the M matrices in these equations. For example, $M_{1\theta \pm }$ is given as

$$\begin{array}{c} M_{1\theta \pm }=ik\gamma^{l}\left[\sum_{j=1}^{m-1}\mu_{j}\int_{a_{j}}^{b_{j}}r{Ф}_{\theta -}^{l}{Ф}_{\theta \pm }^{n}dr+\mu_{m}\int_{a_{m}}^{b_{m}}\tilde{r}\xi_{r}{Ф}_{\theta -}^{l}{Ф}_{\theta \pm }^{n}dr\right]\\ \text{for}\;l=0,1,\ldots ,m_{1};\;n=0,1,\ldots ,m_{1}. \end{array}$$ (24)

The first term in Eq. (24) relates to the pipe and additional layers attached to the pipe, and this term is known to be orthogonal.^10^ However, the addition of the outer layer (m) complicates the relationship and currently there is no proof that Eq. (24) is orthogonal, even with the second integral being computed using the stretched coordinate. Accordingly, the implementation of the axial matching conditions will be investigated in Sec. III. Finally, Eqs. (22) and (23) are combined with Eq. (19) to deliver the final system equation:

$$\left[\begin{array}{ccc} -M_{1\theta -} & Q_{3g-}^{T} & 0\\ Q_{1g-} & K_{1g}-K_{2g}-2K_{2g}^{T}+2K_{6g}-\omega^{2}K_{4g} & Q_{2g+}\\ 0 & Q_{4g+}^{T} & -M_{3\theta +} \end{array}\right]\left\{\begin{array}{c} B\\ u_{2}\\ \begin{array}{c} C \end{array} \end{array}\right\}=\left\{\begin{array}{c} M_{1\theta +}A\\ Q_{1g+}A\\ \begin{array}{c} 0 \end{array} \end{array}\right\}.$$ (25)

This equation is solved for the unknown modal amplitudes in regions 1 and 3, as well as the displacement in the central section, once one has specified an incident wave field in vector **A**. In the results that follow, this equation is solved using a finite element mesh that consists of three noded line elements and eight noded quadratic elements.

## III. MODE MATCHING IN BURIED STRUCTURES

The key challenge with the implementation of a hybrid SAFE-FE approach for buried pipes is the implementation of the appropriate boundary conditions for the material that surrounds the pipe. Accordingly, in this section the application of these boundary conditions is studied for scattering from a defect, as well as the reduced problem of a step change in the surrounding material. This is because a step change facilitates the isolation of the relevant axial matching conditions and the problem is similar to the one studied by Vogt *et al.*^20^ Note that the accuracy of this method is investigated here for larger pipelines over a relatively large frequency range. This is to maintain relevance to common engineering applications; however, this precludes the use of commercial software to validate these predictions because, as discussed in the introduction, this approach would require a prohibitive number of degrees of freedom.

### A. Scattering from a step change

Vogt *et al.*^20^ examined scattering from a step change between an unburied and a buried cylindrical steel rod, with a diameter of 0.5 mm. This is a rather thin structure and for this reason it is not well suited to testing the convergence of the hybrid method, and so the much larger 8 in. schedule 40 steel pipe studied by Duan *et al.*^15^ is studied here instead. This pipe has an outer radius of 109.54 mm and a wall thickness of h=8.179 mm; the material properties of steel are $c_{T_{1}}=3260\;m/s$ and $\rho_{1}=8030\;\text{kg}/m^{3}$. It is worthwhile retaining some of the geometrical features of the viscometer studied by Vogt *et al.* such as the simple step change from unburied to buried pipe; however, the surrounding material should be more representative of that typically found in pipeline applications and so two common materials are examined here:^15^ dry sand with $c_{T_{m}}=105\;m/s$ and $\rho_{m}=1620\;\text{kg}/m^{3}$, and fine soil with $c_{T_{m}}=300\;m/s$ and $\rho_{m}=2000\;\text{kg}/m^{3}$. The step change is assumed to extend to infinity in the radial direction and this is modelled using a PML with values of α=4 and β=4 [see Eq. (4)]. These values for the PML have been chosen following extensive parametric studies aimed at minimising the thickness of the PML in order to reduce solution times. The parametric study is similar in nature to that undertaken by Duan and Kirby^15^ for the eigenproblem, and the general observations drawn by Duan and Kirby are applicable also to this current model. Moreover, this article focuses on the scattering of leaky modes and this permits the PML to be attached directly to the outer surface of the pipe. This is demonstrated by Duan and Kirby,^15^ and it is also further demonstrated for the scattering problem later on in Sec. III B of this article. It should be noted, however, that for different problems, such as those where surface waves are of interest, attaching a PML layer directly to the outer surface of the pipe may produce non-physical predictions. This can easily be addressed by adding an additional layer between the pipe and the PML. However, in this article the focus remains on the scattering of leaky modes, and following a number of studies into the convergence of the model for leaky modes it is found to be possible to attach the PML to the pipe wall and to reduce the width of the PML (see also Ref. 15) to that of the width of the pipe wall, so that $h=b_{1}-a_{1}$. Clearly, minimising the degrees of freedom required for the PML confers significant computational advantages, especially when one is studying the scattering problem.

The purpose of this article is to introduce a hybrid SAFE-FE method for a buried pipe and so to validate this approach for a step change, a central FE section is retained. That is, region 2 surrounds the step change so that $\Gamma_{A}$ cuts the pipe *in vacuo*, and $\Gamma_{B}$ cuts the buried section. It is of course also possible to examine a uniform step change using only modal expansions on either side of the step change, and then applying mode matching across the interface. This approach was used by Vogt *et al.*,^20^ who used analytic methods to obtain the longitudinal eigenmodes and then mode matching to enforce the axial continuity conditions, although Vogt *et al.* restricted matching to the cylinder only and this meant that they did not enforce a traction free condition over the free surface of the embedding medium. In this article an alternative approach is used because our focus is on the hybrid method, and so a region surrounding the step is discretised using finite elements, so that region 2 has a finite length. This has the additional advantage of enabling comparison between the implementation of the axial matching conditions for a pipe *in vacuo*, which has been studied before, and a buried pipe, which has not been studied before. Accordingly, a two dimensional mesh is used to discretise the step change. The length of region 2 is L=0.01m, and the centre of the step change is equidistant from $\Gamma_{A}$ and $\Gamma_{B}$.

Scattering from the step change is obtained by exciting the pipe using the fundamental torsional mode T(0,1) only. This means that in Eq. (8) $A^{0}=1$, and $A^{n}=0$ for n>0. The solution is obtained by computing the eigenmodes for each region using Eq. (7), and then substituting them into Eq. (25). At least 21 nodes per wavelength is maintained for frequencies up to and including 100 kHz; this delivers an element size of $e_{p}=0.5\;\text{mm}$ in the pipe, and $e_{s}=0.1\text{mm}$ in the sand or soil, which translates into $p_{\theta 1}=m_{1}=35$, $p_{\theta 3}=199$, and $m_{3}=120$. Note that significantly more modes are used in the buried pipe region than in the unburied pipe region. This is because a large number of radiation modes exist in the buried pipe section and the energy carried by these modes is largely confined to the PML region. Therefore, radiation modes do not contribute to enforcing the axial matching continuity conditions over the pipe wall. Thus, it is only the leaky and quasi-evanescent leaky modes that contribute to enforcing the axial matching conditions. This means that it is necessary to obtain a larger number of modes when analysing the buried pipe region in order to ensure that a sufficient number of leaky and quasi-evanescent leaky modes are present. The terminology “quasi-evanescent” is used here to indicate those modes that have a close correlation to evanescent modes in a lossless system. In the current problem, all modes are complex, however some modes have very small real parts and so they oscillate in the near field of a junction in a fashion similar to evanescent modes in lossless systems. These modes do not fit into the classification of propagating modes that are useful for non-destructive testing in buried pipes, and they are of use here only in enforcing the matching conditions over planes $\Gamma_{A}$ and $\Gamma_{B}$. Accordingly, these modes are termed quasi-evanescent in order to retain a link with their lossless counterparts, but also to acknowledge that they are not imaginary modes. This gives a final system matrix of order 16 394, which takes about 1 s to solve at each frequency. Note that the frequency independent stiffness and mass matrices need to be calculated only once, and they are then stored for subsequent calculations at all the other frequencies.

**FIG. 3.**
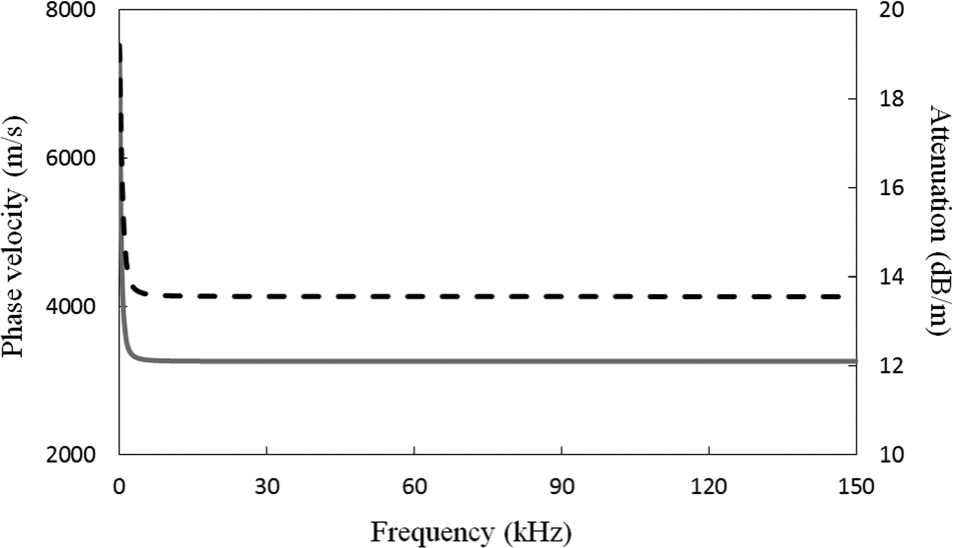
Dispersion curves for T(0,1) in an 8 in. schedule 40 pipe buried in soil: ——, phase velocity; – – – attenuation.

Before the scattering analysis, it is helpful to review the characteristics associated with the incident mode T(0,1), and so phase velocity and attenuation curves for the 8 in. schedule 40 pipe buried in soil are presented in Fig. 3. It can be seen that above 10 kHz, the phase velocity and attenuation curves are non-dispersive, however, as the frequency approaches zero, the energy carried by T(0,1) transfers from the pipe to the soil, and this so this mode converts from a leaky type mode to a radiation type mode and the attenuation increases rapidly. Note that in the frequency range studied here, there is only one propagating torsional mode.

**FIG. 4.**
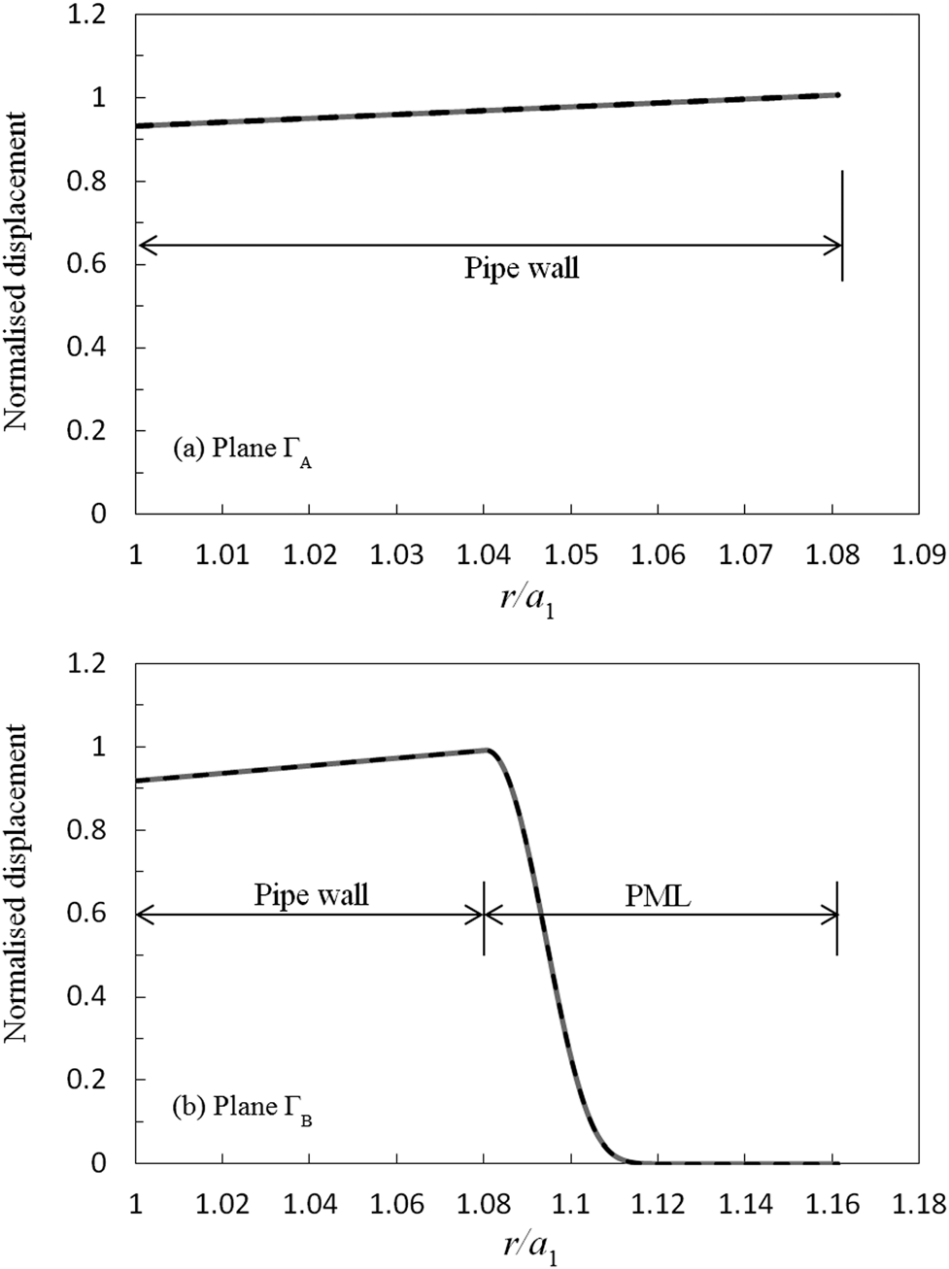
Circumferential displacement for a step change at 50 kHz: ——, modal expansion; – – –, FE solution; (a) plane $\Gamma_{A}$; (b) plane $\Gamma_{B}$: modal expansion overlays FE solution.

**FIG. 5.**
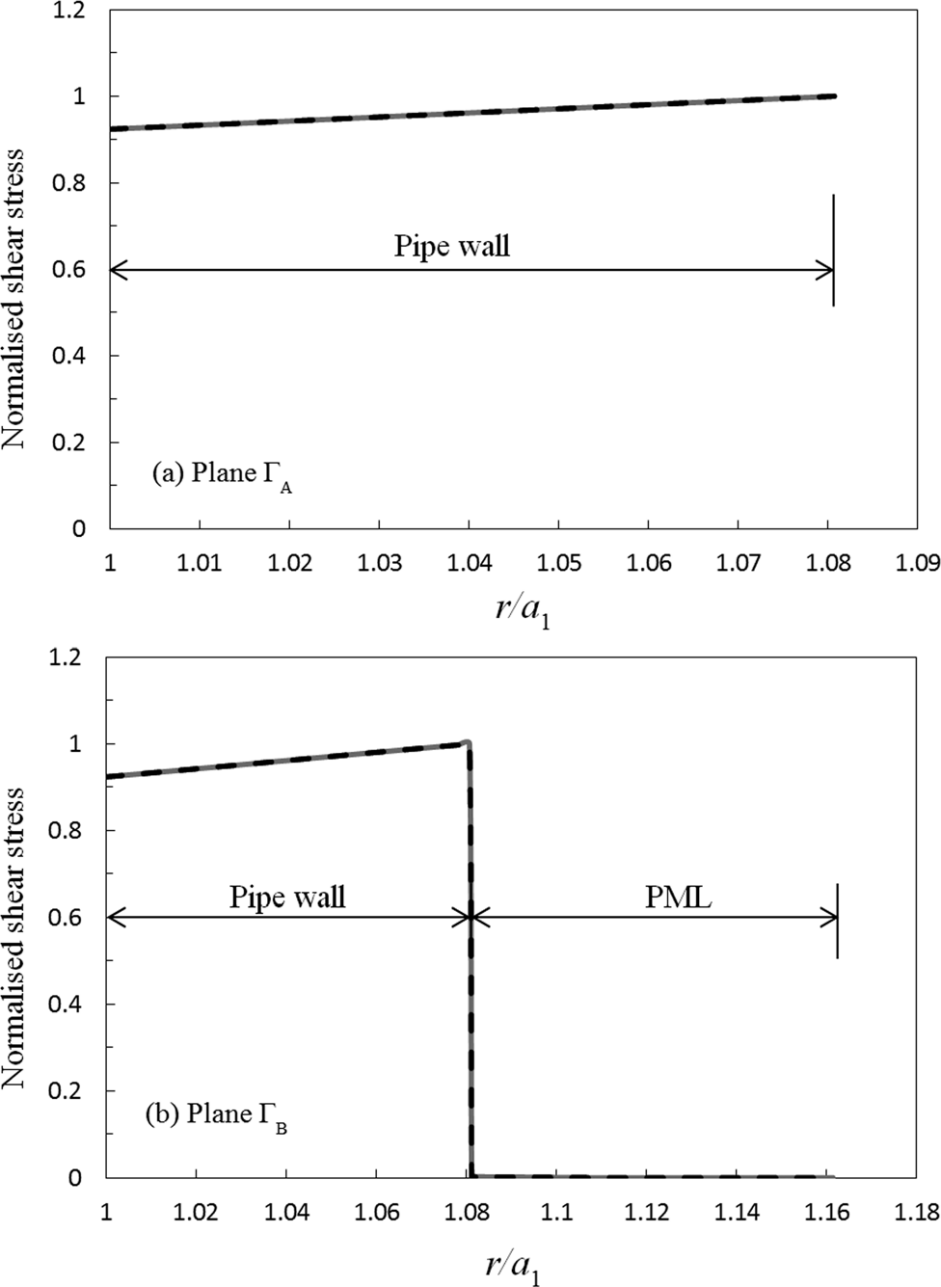
Axial shear stress for a step change at 50 kHz: ——, modal expansion; – – –, FE solution; (a) plane $\Gamma_{A}$; (b) plane $\Gamma_{B}$. Modal expansion overlays FE solution.

There are no alternative analytic or numerical solutions currently available in the literature for this scattering problem, and so the accuracy of the solution presented here is investigated by examining the implementation of the physical boundary conditions. The implementation of the axial matching conditions is examined first, and in Fig. 4 the normalised circumferential displacement for soil on either side of $\Gamma_{A}$ and $\Gamma_{B}$ is compared for an excitation frequency of 50 kHz. In Fig. 5, this comparison is repeated for the normalised axial shear stress $\sigma_{\theta z}^{\prime }$. It is evident in Figs. 4 and 5 that the axial matching conditions closely match one another and that good agreement has been obtained for both displacement and shear stress. Moreover, if one compares the discrete values calculated at each node over the entire surface, then it is possible to calculate a mean average relative error for each matching condition. For $\Gamma_{A}$ this yields a mean error of $10^{-12}$ for displacement, and $10^{-5}$ for shear stress; for $\Gamma_{B}$ the mean error is $10^{-7}$ for displacement and $10^{-5}$ for shear stress. This demonstrates that the accuracy achieved when enforcing the axial continuity conditions is satisfactory even when one includes the surrounding medium. This is important, because in the unburied section the modes are known to be orthogonal, whereas in the buried section Eq. (24) delivers only a semi-orthogonality relation. It is seen in Figs. 4 and 5 that the absence of a true orthogonality relation has not unduly affected the ability to enforce the axial matching conditions in the buried section. A further test of this semi-orthogonality condition may be obtained by looking at the size of the off-diagonal elements in Eq. (24), and here the values on the first row (and column) are $O\left(10^{-8}\right)$, whereas the values for all other off diagonal elements are $O\left(10^{-3}\right)$, for both soil and sand. These values are similar to those observed by Vogt *et al.*,^20^ and so when combined with the results in Figs. 4 and 5 it appears reasonable to conclude that the semi-orthogonality relation in Eq. (24) provides a convergent system of equations. This has been further verified by repeating these tests for many different parameters, including for different pipe sizes. It is interesting also to observe that the PML is highly efficient in absorbing sound power, so that in Figs. 4 and 5 the displacement on the outer surface of the PML is seen to be very close to zero, even for a very modest PML thickness. This ensures that no artificial reflections arise from the PML at the outer boundary, so that the radial boundary condition is properly enforced. Accordingly, the results presented here provide confidence in extending the hybrid SAFE-FE method to buried systems and shows that it is possible to use a one-dimensional PML based numerical mode matching approach to accommodate infinite systems in the radial direction.

It should be noted here that the level of accuracy seen in Figs. 4 and 5 is possible only after the use of appropriate quasi-evanescent modes in the modal expansions that appear in Eqs. (8) and (9). In this application, seven so-called “leaky”^15^ quasi-evanescent modes were required to achieve high levels of accuracy in the implementation of the axial matching conditions. The number required is likely to vary with frequency and other parameters but they need to be included for most practical frequencies of interest. This makes the use of analytic mode matching schemes very difficult for this type of problem, as it is likely to be extremely challenging to obtain appropriate numbers of quasi-evanescent leaky modes from analytic dispersion relations. That is, for scattering problems numerical methods are attractive because they readily calculate those modes necessary for implementing mode matching schemes. Note that Vogt *et al.*^20^ did not encounter problems when using analytic solutions of the dispersion relation because they did not match over the entire step change. Restricting matching to the central structure means that the singularity in the stress field at $r=b_{1}$ disappears, and so Vogt *et al.* were able to generate a convergent system of equations using only a small number of propagating modes. However, if one seeks also to include the zero axial stress boundary condition over the free surface of the step, then the singularity re-appears. In order to overcome this it is necessary to include quasi-evanescent leaky modes and the number required is related to the strength of the singularity.^21^ Accordingly, the analysis of scattering problems of the type studied here inevitably leads toward the use of numerical methods, and especially those methods that prioritise fast and efficient solutions of the eigenproblem.

**FIG. 6.**
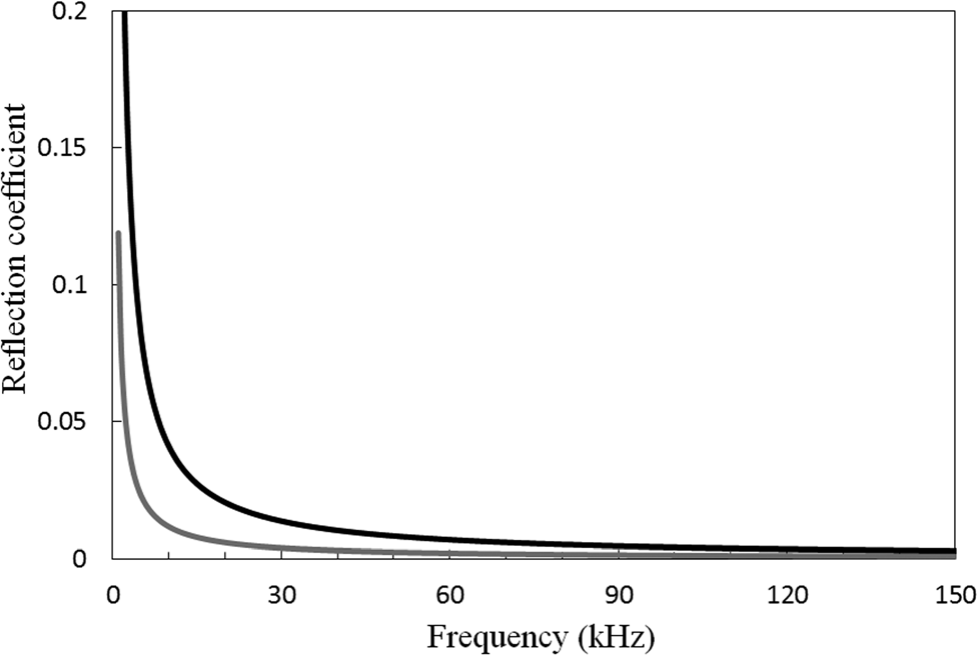
Reflection coefficient from a step change. ——, soil; ——, dry sand.

Vogt *et al.*^20^ went on to calculate reflection coefficients, and these can readily be calculated here through the use of the modal amplitudes obtained on solution of Eq. (25). Accordingly, the reflection coefficient for a step change, which is defined as $B^{0}/A^{0}e^{ik\gamma^{0}L}$, is shown in Fig. 6, for both sand and soil. It is seen in Fig. 6 that the reflection coefficient follows behaviour similar to that seen by Vogt *et al.*,^20^ so that at lower frequencies the reflection coefficient tends toward unity, whereas at higher frequencies it tends toward zero. This is because modal energy of T(0,1) is switching from pipe to soil and this mode is converting from a leaky mode to a radiation mode as the frequency approaches zero. Moreover, it is also seen that soil has a higher reflection coefficient than sand due to the higher acoustic impedance of the soil. Figure 4 also indicates that below about 30 kHz the embedding medium will start reflecting significant levels of energy, and if one is looking to overcome problems with attenuation in buried structures, problems may arise if the strategy is to steadily lower the excitation frequency. Accordingly, in the practical application of LRUT it may be prudent to cut away the surrounding material at a shallow angle rather than leave a steep step change such as the one studied here.

### B. Scattering from a defect

This section examines the more difficult problem of scattering from a defect. The defect is first chosen to be uniform so that the problem is similar to the one studied by Kirby *et al.*^22,23^ Accordingly, a 3 in. schedule 40 steel pipe is examined, with an outer radius of $b_{1}=$ 44.65 mm and a wall thickness of 5.65 mm. The defect is uniform in the axial direction and rectangular (so that φ=90°) with an inner radius of $a_{2}=$ 41.85 mm, and a length of $l_{d}=15\;\text{mm}$ (see Fig. 1). The distance between planes $\Gamma_{A}$ and $\Gamma_{B}$ and the defect is $l_{e}=$ 5 mm. The material properties of the steel pipe and the surrounding dry sand are the same as in Sec. III A. The thickness of the PML is equal to the thickness of the pipe wall, so that h=5.65 mm. The element size in the pipe is $e_{p}=0.5\text{mm}$, and in the sand $e_{s}=0.1\;\text{mm}$, which ensures that at least 21 nodes per wavelength is maintained at the upper frequency of 100 kHz. The solution of Eq. (7) with $m_{1}=m_{3}=120$ delivers a final system matrix of order 67 534, and this takes about 2 s to solve at each frequency.

**FIG. 7.**
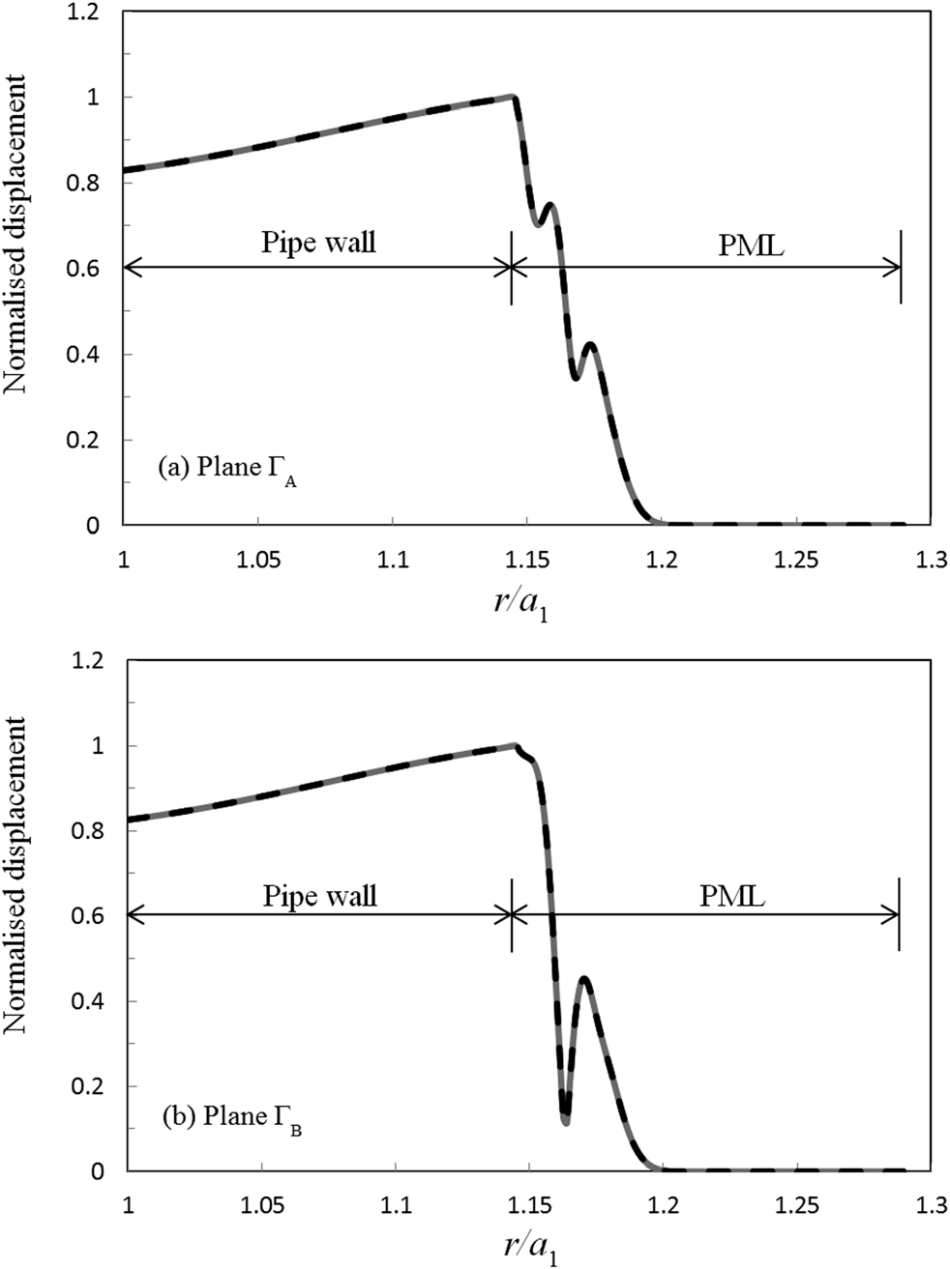
Circumferential displacement for a uniform defect at 100 kHz: ——, modal expansion; – – –, FE solution; (a) plane $\Gamma_{A}$; (b) plane $\Gamma_{B}$. Modal expansion overlays FE solution.

**FIG. 8.**
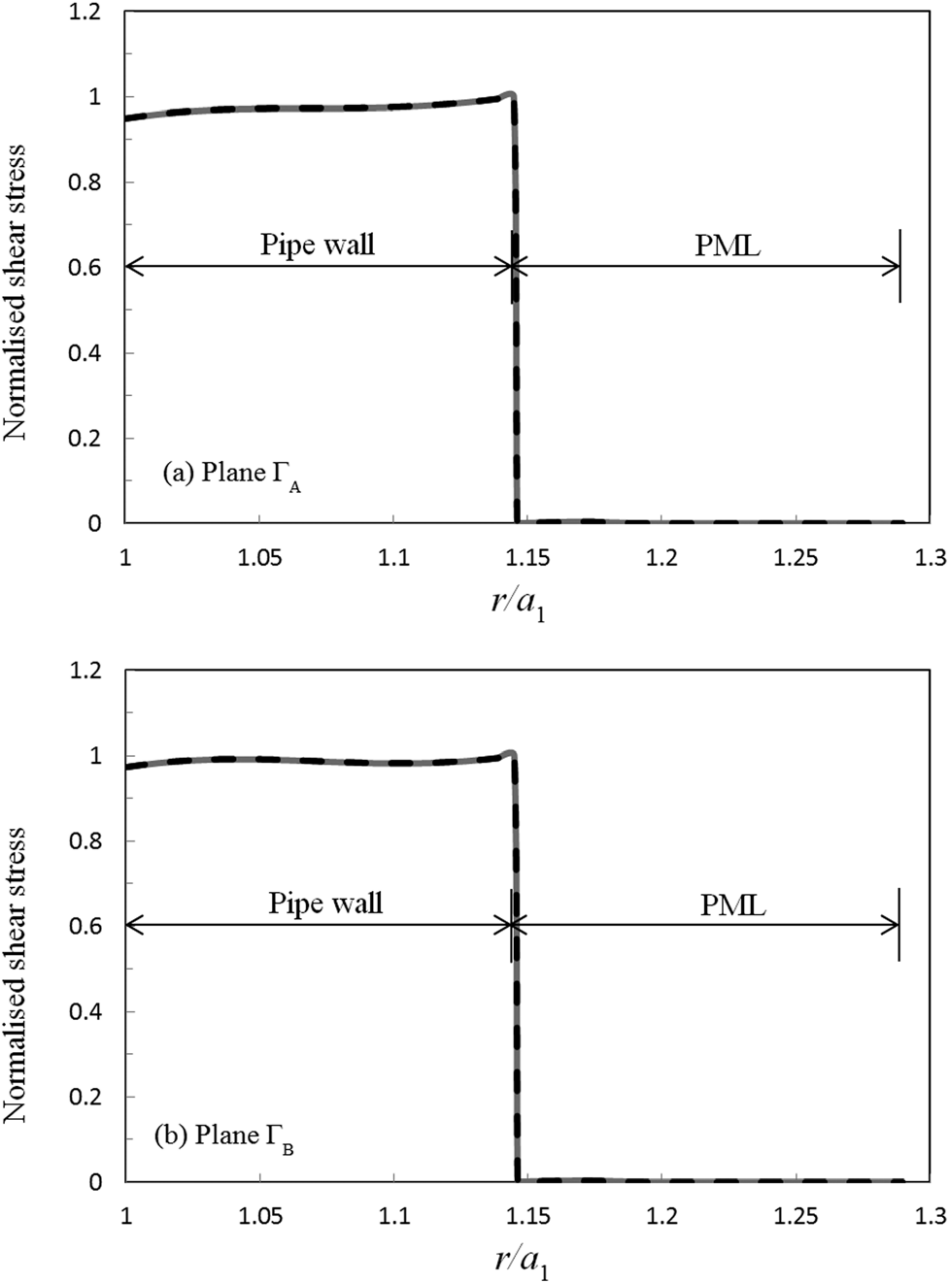
Axial shear stress for a uniform defect at 100 kHz: ——, modal expansion; – – –, FE solution; (a) plane $\Gamma_{A}$; (b) plane $\Gamma_{B}$. Modal expansion overlays FE solution.

The implementation of the matching conditions is examined for a uniform defect in the same way as for the step change in the previous section. Accordingly, in Figs. 7 and 8 those solutions obtained for displacement and shear stress over planes $\Gamma_{A}$ and $\Gamma_{B}$ using modal expansions in regions 1 and 3, are compared to FE based solutions in region 2. It can be seen that the modal expansions again match the FE solutions over both planes, and this yields a mean average relative error of $10^{-10}$ for displacement and $10^{-5}$ for the shear stress for both $\Gamma_{A}$ and $\Gamma_{B}$. Thus, the axial matching conditions are seen to be fulfilled accurately for this scattering problem and this provides further evidence that the hybrid SAFE-FE method may be extended to the study of defects of a finite length. Furthermore, the radial displacement is again seen to quickly go to zero in the outer section, and this further demonstrates the efficiency of the PML based approach.

**FIG. 9.**
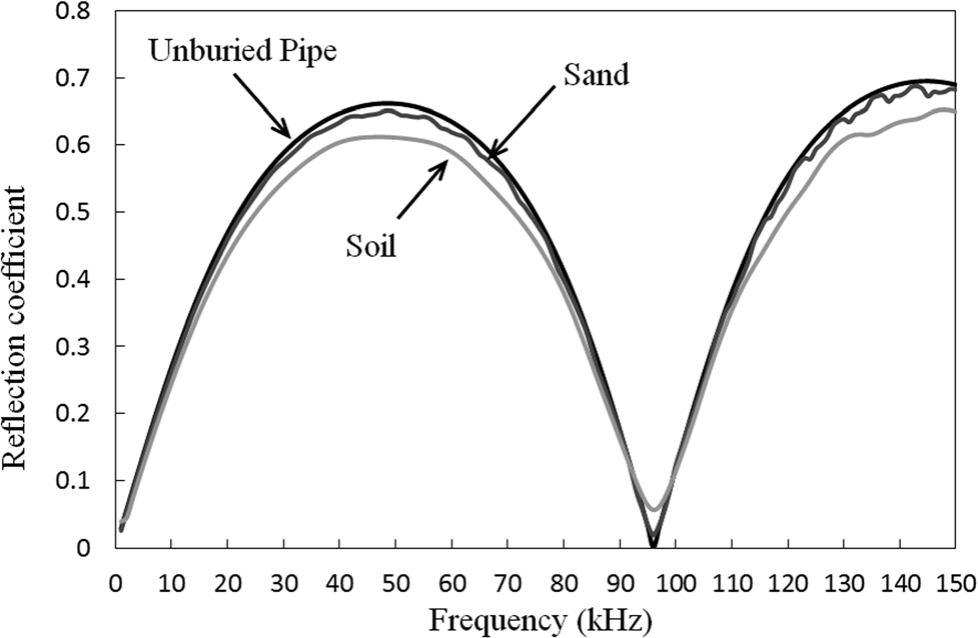
Reflection coefficient for uniform defect buried in sand or soil. Dark line, unburied pipe; medium line, sand; light line, soil. Numerical experiments (a) to (e) in Table I overlay one another for both sand and soil.

The reflection coefficient for the uniform defect may be calculated in the same way as that for the step change; however, region 1 is now buried and so propagating waves in this section will be attenuated as they travel along the pipe. Accordingly, in order to avoid the influence of an arbitrary length of buried pipe, the reflection coefficient for the uniform defect is calculated at the axial position $z=l_{e}$. In Fig. 9 the reflection coefficient for a uniform defect is shown for a pipe buried in dry sand and soil. To reveal the effects of the sand or soil on scattering by the defect, the reflection coefficients obtained are also compared against an equivalent value calculated for an unburied pipe.^22^ It is seen that the reflection coefficient for a buried pipe is slightly lower than that for an unburied pipe. This is because sound energy radiates from the pipe into the surrounding medium, although the amount of energy radiated is small because the impedance of the dry sand or soil is much lower than the impedance of the pipe. However, the dry sand or soil does facilitate axial resonances between the walls of the defect, which is seen to trap small amounts of energy and this shows up as oscillations in the reflection coefficient in Fig. 9. The shear impedance of soil is also larger than in dry sand, and so more energy radiates into the soil than into the sand.

To further explore the relative convergence of the finite element model to a unique solution over a wide frequency range, a number of parameters that are important in determining the accuracy of the model are also investigated here. This includes the element density in the pipe $e_{p}$, the element density in the sand or soil $e_{s}$, the PML inner radius $a_{m}$, the PML thickness h, and the distance between plane $\Gamma_{A}$ (or $\Gamma_{B}$) and the defect $l_{e}$. A total of five different investigations are listed in Table I, and these are compared against one another in Fig. 9. It is evident here that the reflection coefficients calculated for each scenario overlay one another throughout the frequency range and this further illustrates that as the number of elements are increased the method converges to a unique solution, even over a wide range of parameters.

**TABLE I.** Variation of parameters for uniform defect.

	$a_{m}$	$e_{p}$ (mm)	$e_{s}$ (mm)	h (mm)	$l_{e}$ (mm)
a	$b_{1}$	0.5	0.1	5.65	5
b	$b_{1}$	0.2	0.05	5.65	5
c	$b_{1}$	0.5	0.1	11.3	5
d	$b_{1}$	0.5	0.1	5.65	30
e	$b_{1}+2.5\;\text{mm}$	0.5	0.1	5.65	5

## IV. SCATTERING FROM A NON-UNIFORM DEFECT

In this section some further results are presented for a non-uniform defect, as the study of non-uniform problems is important in generating a model that works for practical problems such as the detection of cracks or regions of corrosion. Accordingly, a tapered defect is chosen here, as this has been studied before in the literature for unburied pipes.^22,23^ The 8 in. schedule 40 steel pipe seen in Sec. III A is used and the defect has a length of $l_{d}=38.89$ mm and a taper angle φ=30°, so that the inner radius of the defect is a=104.087  mm. The thickness of the defect (i.e., $b_{1}-a$) is two thirds of the pipe wall. An element size of 0.5 mm is used in the pipe, and 0.1 mm in the surrounding medium, which is either soil or dry sand (see previous section for relevant properties). The PML layer is attached directly to the outer surface of the pipe, with $h=b_{1}-a_{1}$, and $l_{e}=$ 5 mm.

**FIG. 10.**
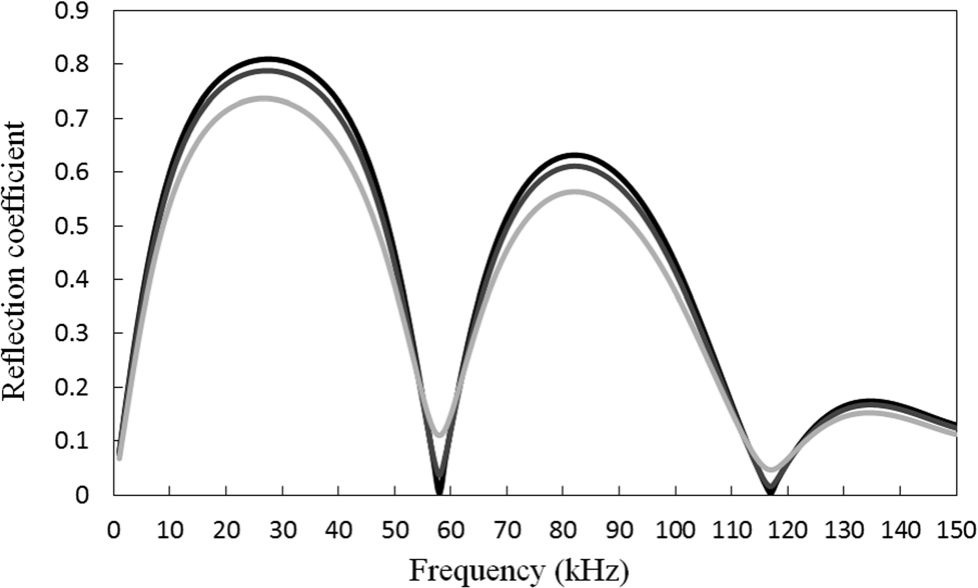
Reflection coefficient for a non-uniform (tapered) defect. Dark line, unburied pipe; medium line, sand; light line, soil.

**FIG. 11.**
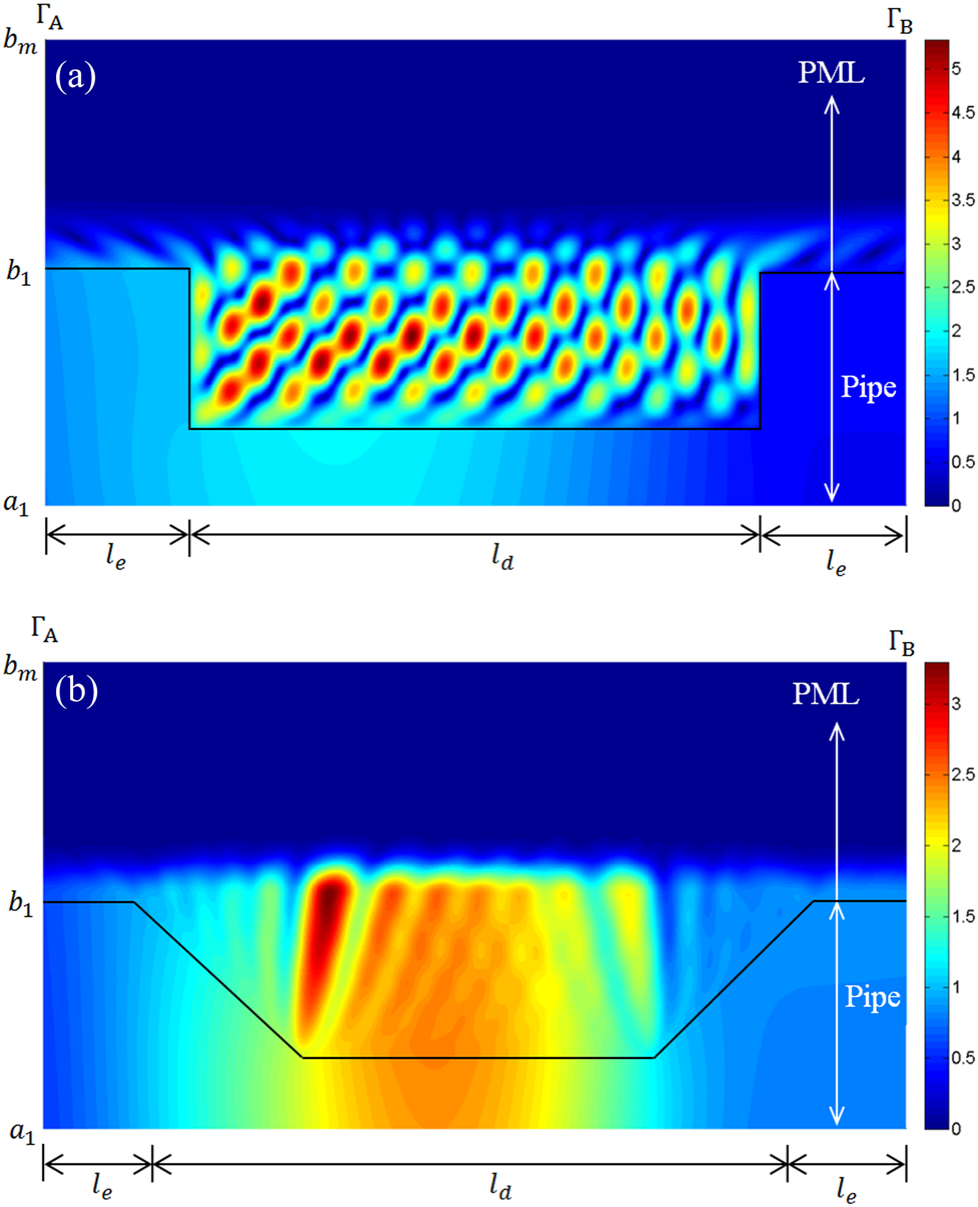
(Color online) (a) Normalised displacement for a uniform defect at 50 kHz. (b) Normalised displacement for non-uniform defect at 50 kHz.

In Fig. 10 the reflection coefficient is shown for T(0,1) incident upon the tapered defect. The reflection coefficient is calculated at $z=l_{e}$ in the same way as Sec. III B, so that the influence of sound attenuation in the pipe is removed. A comparison between Figs. 9 and 10 reveals that the reflection coefficient for the tapered defect is smooth and does not exhibit the small oscillations seen for the uniform defect. This is because the tapering of the defect removes the resonance field between the two walls of the uniform defect and so energy is radiated away from the defect and into the surrounding medium. This causes the reflection coefficient to drop for the tapered defect when compared to the uniform defect. To further illustrate this effect, the displacement field is shown for a uniform defect buried in sand in Fig. 11(a), and a non-uniform defect buried in sand in Fig. 11(b). Here it is seen that tapering removes the resonant behaviour associated with a uniform defect, which is to be expected. Moreover, the surfaces of the taper provide a larger area over which to radiate energy into the surrounding medium and this is why the reflection coefficient is lower for the tapered defect when compared to the uniform defect. This means that regions of corrosion that have geometries similar to the tapered defect studied here are likely to be more difficult to detect in buried structures when compared to unburied structures.

## V. CONCLUSIONS

The theoretical analysis of scattering from defects in buried structures is a challenging problem, which requires a bespoke computational approach in order to deliver a computationally efficient and tractable solution. This article presents a method for doing this which makes use of a hybrid approach based on a SAFE method for obtaining the eigenmodes in a uniform buried section, and then couples this to an FE discretisation of a non-uniform section that surrounds the buried defect. In view of the difficult nature of this problem, the analysis is restricted here to the development of a model for an axisymmetric defect with excitation by torsional modes only.

The results presented in this article demonstrate that the hybrid SAFE-FE model can be successfully applied to an axisymmetric scattering problem. It is seen that mode matching may be used to join the uniform and non-uniform regions together, and that this approach accurately enforces the axial matching conditions provided a significant number of quasi-evanescent leaky modes are included. This is the case even though the eigenmodes in the buried section are only semi-orthogonal. Furthermore, through an appropriate choice of a PML for the surrounding region, and by integrating over the stretched co-ordinate in this region, it is shown that it is possible simultaneously to enforce the appropriate radial and axial boundary conditions in the embedding medium. That is, the hybrid method can be extended to the analysis of buried structures, at least for the axisymmetric torsional problem.

The hybrid method presented here removes the need to discretise the entire length of a structure, and so demonstrates that it is possible to develop efficient theoretical models suitable for analysing scattering in buried structures. Furthermore, the speed of solution at each frequency is less than about 2 s for the problems studied here, which means that the method can readily be extended to the time domain using Fourier Transforms,^9,10^ and the authors have already obtained time domain predictions for this problem which have not been presented here to save space. However, the analysis of guided waves in buried structures remains a complex problem so that the analysis reported here represents a first step toward tackling more difficult three dimensional problems. Accordingly, future work will seek to advance this current model and to include longitudinal and flexural modes, as well as non-axisymmetric defects.

## APPENDIX A

For layers j=1 to m,

$$K_{1j}=\int_{a_{j}}^{b_{j}}\frac{1}{\xi_{r}}\frac{\partial N^{T}}{\partial r}\frac{\partial N}{\partial r}dr,$$ (A1)

$$K_{2j}=\int_{a_{j}}^{b_{j}}\frac{1}{\tilde{r}}\frac{\partial N^{T}}{\partial r}Ndr,$$ (A2)

$$K_{4j}=\int_{a_{j}}^{b_{j}}\xi_{r}N^{T}Ndr,$$ (A3)

$$K_{6j}=\int_{a_{j}}^{b_{j}}\frac{\xi_{r}}{{\tilde{r}}^{2}}\;N^{T}Ndr,$$ (A4)

where for j=1 to m−1, $\xi_{r}=1$, and $\tilde{r}=r$. And

$$Z_{1\theta }=\sum_{j=1}^{m-1}\mu_{j}\left[K_{1j}-K_{2j}-2K_{2j}^{T}+2K_{6j}-\frac{\rho_{j}\omega^{2}}{\mu_{j}}K_{4j}\right],$$ (A5)

$$Z_{1m}=\mu_{m}\left[K_{1m}-K_{2m}-2K_{2m}^{T}+2K_{6m}-\frac{\rho_{m}\omega^{2}}{\mu_{m}}K_{4m}\right].$$ (A6)

## APPENDIX B

$$\begin{array}{c} K_{1g}=\sum_{j=1}^{m-1}\mu_{j}\int_{\Omega_{j}}\nabla N_{2}^{T}\cdot \nabla N_{2}d\Omega_{j}\\ +\mu_{m}\int_{\Omega_{m}}\left[\frac{1}{\xi_{r}}\frac{\partial N_{2}^{T}}{\partial r}\frac{\partial N_{2}}{\partial r}+\xi_{r}\frac{\partial N_{2}^{T}}{\partial z}\frac{\partial N_{2}}{\partial z}\right]\;d\Omega_{m} \end{array},$$ (B1)

$$K_{2g}=\sum_{j=1}^{m-1}\mu_{j}\int_{\Omega_{j}}\frac{1}{r}\frac{\partial N_{2}^{T}}{\partial r}N_{2}d\Omega_{j}+\mu_{m}\int_{\Omega_{m}}\frac{1}{\tilde{r}}\frac{\partial N_{2}^{T}}{\partial r}N_{2}d\Omega_{m},$$ (B2)

$$K_{4g}=\sum_{j=1}^{m-1}\rho_{j}\int_{\Omega_{j}}N_{2}^{T}N_{2}d\Omega_{j}+\rho_{m}\int_{\Omega_{m}}\xi_{r}N_{2}^{T}N_{2}d\Omega_{m},$$ (B3)

$$K_{6g}=\sum_{j=1}^{m-1}\mu_{j}\int_{\Omega_{j}}\frac{1}{r^{2}}N_{2}^{T}N_{2}d\Omega_{j}+\mu_{m}\int_{\Omega_{m}}\frac{\xi_{r}}{{\tilde{r}}^{2}}\;N_{2}^{T}N_{2}d\Omega_{m},$$ (B4)

$$\begin{array}{c} Q_{1g\pm }=ik\gamma^{n}\left[\sum_{j=1}^{m-1}\mu_{j}\int_{a_{j}}^{b_{j}}N_{2}^{T}{Ф}_{\theta \pm }^{n}dr+\mu_{m}\int_{a_{m}}^{b_{m}}\xi_{r}\;N_{2}^{T}{Ф}_{\theta \pm }^{n}dr\right],\\ n=1,2\ldots m_{1} \end{array}$$ (B5)

$$\begin{array}{c} Q_{2g+}=ik\beta^{n}\left[\sum_{j=1}^{m-1}\mu_{j}\int_{a_{j}}^{b_{j}}N_{2}^{T}\;\Psi_{\theta +}^{n}dr+\mu_{m}\int_{a_{m}}^{b_{m}}\xi_{r}N_{2}^{T}\Psi_{\theta +}^{n}dr\right],\\ n=1,2\ldots m_{3} \end{array}$$ (B6)

$$\begin{array}{c} Q_{3g-}=ik\gamma^{n}\left[\sum_{j=1}^{m-1}\mu_{j}\int_{a_{j}}^{b_{j}}rN_{2}^{T}\;{Ф}_{\theta -}^{n}dr+\mu_{m}\int_{a_{m}}^{b_{m}}\tilde{r}\xi_{r}\;N_{2}^{T}{Ф}_{\theta -}^{n}dr\right]\;(n=0,1,\ldots ,m_{1}) \end{array}$$ (B7)

$$\begin{array}{c} Q_{4g+}=ik\beta^{n}\left[\sum_{j=1}^{m-1}\mu_{j}\int_{a_{j}}^{b_{j}}rN_{2}^{T}\;\Psi_{\theta +}^{n}dr+\mu_{m}\int_{a_{m}}^{b_{m}}\tilde{r}\xi_{r}\;N_{2}^{T}\Psi_{\theta +}^{n}dr\right]\;(n=0,1,\ldots ,m_{3}) \end{array},$$ (B8)

$$\begin{array}{c} M_{1\theta \pm }=ik\gamma^{l}\left[\sum_{j=1}^{m-1}\mu_{j}\int_{a_{j}}^{b_{j}}r{Ф}_{\theta -}^{l}{Ф}_{\theta \pm }^{n}dr+\mu_{m}\int_{a_{m}}^{b_{m}}\tilde{r}\xi_{r}{Ф}_{\theta -}^{l}{Ф}_{\theta \pm }^{n}dr\right](l=0,1,\ldots ,m_{1};n=0,1,\ldots ,m_{1}) \end{array},$$ (B9)

$$\begin{array}{c} M_{3\theta +}=ik\beta^{l}\left[\sum_{j=1}^{m-1}\mu_{j}\int_{a_{j}}^{b_{j}}r\Psi_{\theta +}^{l}\;\Psi_{\theta +}^{n}dr+\mu_{m}\int_{a_{m}}^{b_{m}}\tilde{r}\xi_{r}\Psi_{\theta +}^{l}\Psi_{\theta +}^{n}dr\right]\\ (l=0,1,\ldots ,m_{3};n=0,1,\ldots ,m_{3}). \end{array}$$ (B10)
